# Comparison of Two Different Scheimpflug Devices in the Detection of Keratoconus, Regular Astigmatism, and Healthy Corneas

**DOI:** 10.1155/2015/315281

**Published:** 2015-04-14

**Authors:** David Finis, Bernhard Ralla, Maria Karbe, Maria Borrelli, Stefan Schrader, Gerd Geerling

**Affiliations:** Department of Ophthalmology, Heinrich-Heine-University Düsseldorf, Moorenstraße 5, 40225 Düsseldorf, Germany

## Abstract

*Aim*. The aim of this study was to determine the intra- and interobserver variability of two Scheimpflug based camera systems, Pentacam and Sirius. In addition, the comparability of the measurements was tested in healthy subjects, subjects with regular astigmatism, and keratoconus patients.* Methods*. Intra- and interobserver variability were assessed in 20 healthy corneas. Pachymetry values were also compared with ultrasound pachymetry as a reference measurement. To detect possible differences between the two devices, 82 eyes with clinically established keratoconus, 30 eyes with regular astigmatism (>1.5 D), and 60 eyes without corneal pathologies were included in this prospective study.* Results*. Pachymetry and keratometry showed good intra- and interobserver variability for both devices. Pachymetry values obtained with the Sirius system (579 ± 58 *μ*m) were significantly higher compared to the Pentacam system (551 ± 40 *μ*m, *P* < 0.001) and to ultrasound pachymetry (550 ± 43 *μ*m, *P* < 0.001). Significant interdevice differences were found regarding the majority of the detected keratometry parameters.* Conclusions*. Both devices show almost perfect reproducibility in healthy subjects. However, pachymetry and keratometry values of the two devices should not be used interchangeably.

## 1. Introduction

Keratoconus is a bilateral and chronic progressive disease. Due to different definitions and diagnostic criteria prevalence varies between 4 and 60/100.000 affected patients. Histological findings include stromal thinning, iron deposition in the epithelial basement membrane, and breaks in Bowman's layer. The etiology and pathogenesis of keratoconus is still unknown but may be associated with a variety of factors like eye rubbing, Down syndrome, atopic disease, connective tissue disease, or tapetoretinal degeneration [1–5].

Although keratoconus can show several typical clinical signs like corneal thinning, irregular astigmatism, or Vogt-Striae an early diagnosis can be challenging. On the other hand an early diagnosis becomes more important since with corneal cross-linking a promising treatment to slow or even to stop the progression of keratoconus has been established as first described by Wollensak et al. In addition post-LASIK corneal ectasia is still one of the most feared complications of refractive surgery. A major risk factor for the development is forme fruste keratoconus which makes an early detection even more important [6–9].

Probably the best suitable devices for early detection of keratoconus are Scheimpflug based cameras. Before development and clinical introduction of this technology, corneal imaging was limited to the analysis of the anterior corneal surface. Scheimpflug based systems allow anterior and posterior corneal surface and corneal thickness measurement and are capable of calculating a three-dimensional model of the anterior segment. The newer generations of Scheimpflug based systems are using rotating cameras, wherein the rotation axis is centered on the corneal apex. In contrast to Placido based topography systems, which interpolate the central region because of the location of the camera, the rotating Scheimpflug camera systems are capable of delivering exact measurements of the optically important central cornea. There are several different versions of Scheimpflug based systems available. Two of them are the Pentacam (Oculus, Wetzlar, Germany) and the Sirius Scheimpflug Analyzer (CSO, Costruzione Strumenti Oftalmici, Florence, Italy). While the Pentacam uses only a rotating Scheimpflug camera the Sirius system combines a rotating Scheimpflug camera with a 22-ring Placido disc corneal topographer, to better analyze the anterior corneal curvature [10, 11].

The aim of this study was to determine the intra- and interobserver variability of the two Scheimpflug based camera systems Pentacam and Sirius and to assess the interdevice variability of the measurements in healthy subjects, subjects with regular astigmatism, and keratoconus patients.

## 2. Material and Methods

The study protocol adhered to the tenets of the Declaration of Helsinki and was approved by the ethics board of the Heinrich-Heine-University Düsseldorf. Informed consent was provided by all participants after the nature and possible consequences of the investigation had been explained to them.

We studied the intra- and interobserver variability in 20 eyes of 10 healthy subjects (10 subjects, 3 male and 7 female, age: 24 ± 7 years). All of these eyes were analysed with both devices three times and by three different observers in a randomized sequence. Each time the complete examination was repeated, that is, both the measurement and the analysis with each device. The pachymetry values were also compared with an ultrasound pachymeter as a reference measurement. For statistical analysis of the inter- and intraexaminer variability the intraclass correlation coefficient (ICC) was determined.

To detect the differences between the two devices we included 82 eyes with a long-standing clinical diagnosis of keratoconus (group I: 50 patients, 35 male 15 female, age: 39 ± 14 years), 30 eyes with regular astigmatism >1.5 D (group II: 21 subjects, 14 male 7 female, age: 49 ± 22 years), and 60 eyes without corneal pathology (group III: 33 subjects, 15 male 18 female, age: 43 ± 21 years) in this study. To determine interdevice differences all individual measurements of these 172 eyes were compared.

The inclusion criteria for the three groups ((1) keratoconus patients, (2) patients with regular astigmatism, and (3) control subjects) were as follows:

1. Group I: patients with prediagnosed keratoconus. The diagnosis had to be confirmed by topographical analysis with the two Scheimpflug devices. An asymmetric bow-tie, skewed radial axes or inferior steepening was considered to be a keratoconus-like pattern. In addition the steepest corneal refractive power had to be above 50 D.
2. Group II: subjects without known corneal pathologies with a corneal astigmatism of more than 1.5 D. This had to be confirmed by topographic analysis with both devices.
3. Group III: subjects without known corneal pathologies with a corneal astigmatism of less than 1.5 D. This had to be confirmed by topographic analysis with both devices.

Exclusion criteria were as follows: any other known corneal pathology, any eyelid abnormalities, any history of intraocular surgery, inability, or unwillingness to participate in the study. In addition all patients with keratoconus-like topographical pattern as mentioned above or with a suspicion for forme fruste keratoconus detected by either the Pentacam or the Sirius system were excluded to participate in study group II or III.

Measurements with the Oculus Pentacam and with the CSO Sirius were performed according to manufacturer's instructions. For the present study the Pentacam HR with the software version 1.17 and the Sirius with the software version Phoenix 1.0.5.72 were used. Of the multitude of parameters analysed by both systems we selected the subsequent list:

1. Pachymetry at the apex.
2. Pachymetry at the thinnest point.
3. Flat meridian with three-millimeter distance from the apex.
4. Steep meridian with three-millimeter distance from the apex.
5. Flat meridian with five-millimeter distance from the apex.
6. Steep meridian with five-millimeter distance from the apex.
7. Flat meridian with seven-millimeter distance from the apex.
8. Steep meridian with seven-millimeter distance from the apex.
9. Flat meridian of the corneal back surface radius with three-millimeter distance from the apex.
10. Steep meridian of the corneal back surface radius with three-millimeter distance from the apex.

For the detection of corneal pachymetry we used the Tomey AL-400 ultrasound pachymeter. After topical anaesthesia with (oxybuprocaine hydrochloride 4.0 mg/mL) the central corneal thickness was measured 8 times with automatic release by an experienced observer. The mean value of all measurements was used for further calculations. All ultrasound measurements were performed after topographic analysis.

Statistical analysis was performed using SPSS Statistics 20 for Windows 7. For statistical analysis of the inter- and intraexaminer variability the intraclass correlation coefficient (ICC) was used. Requirement for the intraclass correlation was a normal distribution of the measured values. The comparison of the average ICCs of the inter- and intraexaminer differences of the Pentacam and of the Sirius was performed using Student's *t*-tests. To test the interdevice differences also Student's *t*-tests were performed. Significance was defined as *P* < 0.05.

## 3. Results

Determination of intra- and interobserver variability revealed high ICC values for both devices regarding pachymetry, except for the intraobserver variability of the pachymetry at the apex measured with the Sirius system, which was significantly lower (Table 1). Noteworthy, the pachymetry values at the apex were significantly higher measured with the Sirius system compared to the Pentacam system (579 ± 58 *μ*m versus 551 ± 40 *μ*m, *P* < 0.001). The detection of the pachymetry at the apex with the ultrasound pachymeter revealed comparable values to the Pentacam system but significantly differed from the Sirius system (550 ± 43 *μ*m, *P* = 1.0 versus Pentacam, *P* < 0.001 versus Sirius). Regarding keratometry both devices showed good interobserver (Table 2) and intraobserver variability (Table 3). The average value of all measured ICCs was 0.988 ± 0.012 for the Pentacam and 0.949 ± 0.088 for the Sirius, showing a significantly higher value for the Pentacam measurements (*P* = 0.017).

The interdevice differences regarding keratoconus patients (group 1) are shown in Table 4. All values except the pachymetry of the apex, the keratometry of the flat meridian at 3 mm distance to the apex, and the flat meridian of the corneal back surface were significantly different between the two devices. The interdevice differences regarding patients with regular astigmatism (group 2) are displayed in Table 5. All values except the keratometry of the flat meridian at 3 mm distance to the apex and 5 mm distance to the apex and the steep meridian of the corneal back surface were significantly different between the two devices. The interdevice differences regarding control subjects (group 3) are listed in Table 6. All values except the keratometry of the steep meridian of the corneal back surface were significantly different between the two devices.

## 4. Discussion

The results of the present study demonstrate that both the Pentacam and the Sirius system show good ICCs regarding intra- and interobserver variability. Averaged values for both devices were clearly higher than 0.9 which indicates a good reproducibility in clinical routine. However, the average ICC for the Pentacam was significantly higher which indicates a better reproducibility for this system. These results are in accordance with observations made in other studies that report a high reliability for both devices; however, as far as we are aware no study reported better results for the Pentacam system so far [11–18]. Overall, the small superiority of the Pentacam regarding intra- and interobserver variability found in the present study should be considered restrained since both devices show almost perfect reproducibility. However, the combination of the 22-ring Placido disc corneal topographer and a rotating Scheimpflug camera in the Sirius system [10, 11] did not provide an advantage compared to the Pentacam system in this study.

In addition the results of this study revealed that the Sirius system delivers significantly higher pachymetry values than the ultrasound pachymeter and the Pentacam system. Bayhan et al. also reported a difference between an ultrasound pachymetry and the Sirius system with a mean difference of 17.58 ± 8.13 *μ*m [19]. Maresca et al. reported different corneal thickness values obtained with the Sirius system compared to ultrasound pachymetry [20] like Parra-Colin et al. [11]. In contrast Huang et al. reported that central corneal thickness measured with the Sirius system was in high agreement with the ultrasound pachymetry. They reported narrow 95% limits of agreement, for example, −18.59 to 10.90 *μ*m for the thinnest corneal thickness [13, 14]. However these variations are in the same range compared to the other listed studies or the present study, which leads to the conclusion that pachymetry values of the Sirius system cannot be used interchangeably with other devices, especially ultrasound pachymetry. This could also gain particular importance in glaucoma diagnosis and for evaluation of refractive surgery patients.

Furthermore the results displayed in Tables 4–6 show that almost every parameter measured with the two devices showed significant different values. Also, Parra-Colin et al. reported that the Pentacam provides systematically higher mean values for all parameters. However, besides pachymetry values only keratometry values of the steep meridian were significantly higher with a mean difference of 0.31 diopters [11]. Nasser et al. concluded that Sirius and Pentacam values should not be used interchangeably [21]. Ramirez-Miranda et al. found an interdevice agreement for the anterior radius of curvature but not for maximum anterior and posterior corneal elevation and total higher-order aberrations between Sirius and Pentacam HR [12]. Shetty et al. found significant differences in the measurements between the two devices and the Galilei-Scheimpflug system and concluded that the devices cannot be used interchangeably for anterior segment measurements in keratoconus patients [15]. Wang et al. reported significant differences between Sirius and Pentacam regarding mean keratometry but stated that these differences although significant were below 0.1 diopters which is not clinically meaningful [18]. Overall, the last conclusion seems to be of particular importance. Of course smaller differences, albeit significant, especially below 0.5 diopters, are less relevant in clinical routine. In the present study the interdevice difference was below 0.1 mm or 0.5 dpt for the keratometry values in healthy individuals (Table 6), but especially for keratoconus patients we found higher values.

In contrast to previous studies, this study assessed the differences in healthy subjects and subjects with astigmatism and keratoconus patients. While in healthy subjects the two devices appear to provide nearly comparable keratometry values, in keratoconus patients the values are not comparable.

## 5. Conclusion

In summary the present study demonstrates that both devices show very good intra- and interobserver variability. However, pachymetry values of the two devices should not be used interchangeably. Keratometry values of both devices may provide comparable values in healthy individuals but not in keratoconus patients.

## Figures and Tables

**Table 1:** Intra- and interobserver variability of pachymetry in healthy corneas (ICC = intraclass correlation coefficient).

Pachymetry	ICC	
	Pentacam	Sirius
Interobserver variability at the apex	0.992	0.924
Interobserver variability at the thinnest point	0.991	0.996
Intraobserver variability at the apex	0.979	0.743
Intraobserver variability at the thinnest point	0.978	0.989

**Table 2:** Interobserver variability of keratometry in healthy corneas (ICC = intraclass correlation coefficient).

	ICC	
	Pentacam	Sirius
Flat meridian 3 mm to the apex	0.998	0.995
Steep meridian 3 mm to the apex	0.999	0.993
Flat meridian 5 mm to the apex	0.999	0.997
Steep meridian 5 mm to the apex	0.999	0.994
Flat meridian 7 mm to the apex	0.999	0.998
Steep meridian 7 mm to the apex	0.999	0.994
Back surface flat meridian 3 mm to the apex	0.993	0.971
Back surface steep meridian 3 mm to the apex	0.987	0.968

**Table 3:** Intraobserver variability of keratometry in healthy corneas (ICC = intraclass correlation coefficient).

	ICC	
	Pentacam	Sirius
Flat meridian 3 mm to the apex	0.996	0.988
Steep meridian 3 mm to the apex	0.996	0.975
Flat meridian 5 mm to the apex	0.997	0.993
Steep meridian 5 mm to the apex	0.997	0.983
Flat meridian 7 mm to the apex	0.997	0.995
Steep meridian 7 mm to the apex	0.994	0.982
Back surface flat meridian 3 mm to the apex	0.977	0.933
Back surface steep meridian 3 mm to the apex	0.968	0.911

**Table 4:** Interdevice differences of the two devices in keratoconus patients.

	Pentacam	Sirius	*P* values
	Mean ± SD		
Pachymetry (*µ*m)			
Apex	465.87 ± 63.74	466.70 ± 87.52	0.975
Thinnest point	442.27 ± 71.71	416.82 ± 76.04	<0.0001
Keratometry ant. surface (mm)			
3 mm flat meridian	7.096 ± 0.923	7.012 ± 0.955	0.059
3 mm steep meridian	6.643 ± 0.873	6.440 ± 0.967	<0.0001
5 mm flat meridian	7.406 ± 0.716	7.082 ± 0.863	<0.0001
5 mm steep meridian	6.983 ± 0.722	6.596 ± 0.872	<0.0001
7 mm flat meridian	7.760 ± 0.564	7.203 ± 0.771	<0.0001
7 mm steep meridian	7.347 ± 0.595	6.776 ± 0.796	<0.0001
Keratometry back surface (mm)			
3 mm flat meridian	5.884 ± 0.795	5.938 ± 1.217	0.662
3 mm steep meridian	5.235 ± 0.827	4.745 ± 1.177	<0.0001

**Table 5:** Interdevice differences of the two devices in corneas with regular astigmatism.

	Pentacam	Sirius	*P* values
	Mean ± SD		
Pachymetry (*µ*m)			
Apex	560.45 ± 36.41	590.66 ± 55.62	<0.0001
Thinnest point	554.84 ± 35.50	545.88 ± 33.45	<0.0001
Keratometry ant. surface (mm)			
3 mm flat meridian	7.982 ± 0.374	8.007 ± 0.378	0.654
3 mm steep meridian	7.685 ± 0.344	7.631 ± 0.396	0.002
5 mm flat meridian	8.023 ± 0.372	7.996 ± 0.369	0.343
5 mm steep meridian	7.752 ± 0.359	7.663 ± 0.385	0.002
7 mm flat meridian	8.187 ± 0.430	8.042 ± 0.429	<0.0001
7 mm steep meridian	7.914 ± 0.387	7.750 ± 0.387	<0.0001
Keratometry back surface (mm)			
3 mm flat meridian	6.719 ± 0.377	6.838 ± 0.419	0.001
3 mm steep meridian	6.347 ± 0.363	6.317 ± 0.536	0.587

**Table 6:** Interdevice differences of the two devices in healthy corneas.

	Pentacam	Sirius	*P* values
	Mean ± SD		
Pachymetry (*µ*m)			
Apex	554.83 ± 42.85	588.23 ± 69.03	<0.0001
Thinnest point	549.44 ± 38.34	539.35 ± 45.43	<0.0001
Keratometry ant. surface (mm)			
3 mm flat meridian	7.836 ± 0.318	7.793 ± 0.324	<0.0001
3 mm steep meridian	7.708 ± 0.316	7.661 ± 0.334	<0.0001
5 mm flat meridian	7.878 ± 0.320	7.797 ± 0.323	<0.0001
5 mm steep meridian	7.747 ± 0.314	7.678 ± 0.328	<0.0001
7 mm flat meridian	7.999 ± 0.332	7.830 ± 0.325	<0.0001
7 mm steep meridian	7.852 ± 0.309	7.711 ± 0.322	<0.0001
Keratometry back surface (mm)			
3 mm flat meridian	6.571 ± 0.436	6.669 ± 0.561	<0.0001
3 mm steep meridian	6.295 ± 0.336	6.264 ± 0.429	0.337
